# Ketamine Rescues Hippocampal Reelin Expression and Synaptic Markers in the Repeated-Corticosterone Chronic Stress Paradigm

**DOI:** 10.3389/fphar.2020.559627

**Published:** 2020-09-02

**Authors:** Jenessa N. Johnston, Jonathan S. Thacker, Charissa Desjardins, Brian D. Kulyk, Raquel Romay-Tallon, Lisa E. Kalynchuk, Hector J. Caruncho

**Affiliations:** ^1^ Division of Medical Sciences, University of Victoria, Victoria, BC, Canada; ^2^ Department of Psychology, University of Saskatchewan, Saskatoon, SK, Canada

**Keywords:** reelin, ketamine, antidepressant, mammalian target of rapamycin, synaptosomes, hippocampus, lymphocytes

## Abstract

Depression is the leading cause of disability worldwide, which necessitates novel therapeutics and biomarkers to approach treatment of this neuropsychiatric disorder. To assess potential mechanisms underlying the fast-acting antidepressant actions of ketamine we used a repeated corticosterone paradigm in adult male rats to assess the effects of ketamine on reelin-positive cells, a protein largely implicated in the pathophysiology of depression. We also assessed the effects of reelin and ketamine on hippocampal and cerebellar synpatosomes, and on serotonin transporter clustering in peripheral lymphocytes to determine reelin and ketamine’s impact at the synaptic and peripheral levels. Reelin and ketamine similarly rescue synaptic expression of mTOR and p-mTOR that were decreased by corticosterone. Reelin, but not ketamine, was able to rescue patterns of serotonin transporter clustering in the periphery. These findings display ketamine as a powerful modulator of reelin expression and lend strength to further evaluation of the putative fast antidepressant-like actions of reelin.

## Introduction

Major depressive disorder is currently the leading cause of disability in the world, impacting around 16% of the population throughout their lifetime (Friedrich, 2017). Characterized by a constellation of symptomatology that encompasses mental, physical, emotional and cognitive symptoms, there are difficulties in effective treatment in many cases. After a serendipitous discovery that pharmacotherapies targeting the monoaminergic systems were decreasing depressive symptoms, novel antidepressants were developed exclusively following the monoaminergic hypothesis. However, it is now clear from the widespread use of these therapies that a large portion of the patient population are non-responsive and require extended periods to achieve therapeutic effects, raising concerns that the delay in tangible improvement may increase risk of suicide (McCormack and Korownyk, 2018; Shinohara et al., 2019). Therefore, there is a great interest in developing novel antidepressants that work better and faster, such as in the case of Ketamine, a high-affinity non-competitive NMDA receptor antagonist that, in subanesthetic dosages, produces rapid and long-lasting antidepressant effects in patients with treatment-resistant depression (Zarate et al., 2006; Ibrahim et al., 2011). Traditionally hard to target symptomology such as anhedonia, negative cognitive biases, and suicidal ideation have been successfully treated with this compound, making the unique mechanisms of ketamine a great interest to antidepressant researchers. Zanos et al. (2016) found that the behavioral, electrophysiological, electroencephalographic, and cellular antidepressant effects of ketamine were dependent on the increase in glutamatergic transmission that stimulates a fast, transient activation of the mammalian target of rapamycin (mTOR) pathway in the prefrontal cortex and hippocampus. This activation of mTOR stimulates sustained elevation of synapse-associated proteins through p70S6 kinase (p70S6K), such as postsynaptic density-95 protein (PSD95) and increased surface insertion of the Glutamate A1 (GluA1) subunit of α-amino-3-hydroxy-5-methyl-4-isoxazolepropionic acid (AMPA) receptors, a potentially important mechanism underlying the fast-antidepressant actions of ketamine. Interestingly, Abdallah et al. (2020) have recently shown that systemic injections of the mTOR inhibitor rapamycin unexpectedly prolonged, instead of blocking, the antidepressant effects of ketamine, which suggests that perhaps there are multiple mechanisms involved in the fast-acting antidepressant actions of ketamine.

Reelin is a large extracellular glycoprotein initially expressed by Cajal-Reitzus cells throughout development, where it regulates proper neural migration. After development, reelin is primarily expressed by GABAergic interneuron subtypes in the hippocampus and cortex. In the hippocampus, this glycoprotein plays many important roles in the hippocampus including promoting maturation of newborn granule cells (Bosch et al., 2016), enhancing dendritic spine development and maturation (Niu et al., 2008; Chameau et al., 2009; Hethorn et al., 2015), neurogenesis (Teixeira et al., 2011), and learning and memory (Beffert et al., 2005).

Reelin was first found to be involved in neuropsychiatric disorders when a downregulation of reelin expression was observed in the hippocampus of schizophrenia, bipolar, and major depressive disorder patients (Impagnatiello et al., 1998; Fatemi et al., 2000; Guidotti et al., 2000). We have recently shown that intrahippocampal infusions of reelin have a fast-acting antidepressant-like effect in the repeated corticosterone (CORT) paradigm (Brymer et al., 2020), but the mechanisms by which it rescues the depressive-like effects of CORT are yet unknown. As reelin has been shown to enhance dendritic spine development and maturation in healthy animals, our lab looked toward mechanisms that reelin may have at the synaptic level in this pathophysiological model. Our research in hippocampal synaptosomes has shown that reelin, like ketamine, significantly increases expression of mTOR, phosphorylated-mTOR (p-mTOR) and other related proteins in the hippocampus of corticosterone-treated animals within 30 min (Brymer et al., 2020). Using the repeated CORT model, we have also found that decreases in reelin in the dentate gyrus subgranular zone (SGZ) parallel depressive-like symptomology which is rescued by both traditional and non-traditional antidepressants such as imipramine and the Tumor Necrosis Factor-α (TNF-α) inhibitor etanercept (Lussier et al., 2013; Fenton et al., 2015; Brymer et al., 2018).

Depression does not exclusively impact the central nervous system, with a multitude of studies showing changes in the gut microbiome, immune system and more (reviewed in Dantzer et al., 2008; Kelly et al., 2015), which is why it is essential to study potential peripheral mechanisms of pharmacotherapies in treating this neuropsychiatric disorder. Inflammatory events associated with chronic stress have been shown to play a large role in the pathophysiology of depression (reviewed by Leonard, 2018), demonstrated through upregulation of proinflammatory cytokines and alterations in associated peripheral proteins. Our lab has shown that changes in serotonin transporter (SERT) clustering in lymphocytes in treatment naïve depression patients give indications on potential responses to treatment (Rivera-Baltanas et al., 2015). These changes are paralleled in the CORT animal model for the study of depression, as well as in HRM, indicating reelin as a potential modulator of this clustering (Rivera-Baltanas et al., 2010; Romay-Tallon et al., 2018). The serotonergic system plays an essential role in regulating behaviors associated with depression, such as emotion and sleep (reviewed in Kohler et al., 2016), so it is not surprising to see alterations in this system directly involved in the pathophysiology of depression.

Considering this, we hypothesized that ketamine may rescue reelin expressing cells and have similar effects to reelin at the synaptic and peripheral levels in a depressive-like phenotype inducted through repeated administration of exogenous CORT.

## Materials and Methods

### Animals

Forty adult male Long-Evans rats weighing 200-250 g upon arrival were purchased from Charles River Laboratories (Montreal, Quebec, Canada). Rats were housed individually in clear plastic cages with free access to food and water. The thermally controlled colony was maintained on a 12-h/12-h light/dark cycle. All procedures were approved by the University of Saskatchewan Animal Research Ethics Board and/or the University of Victoria Animal Research Ethics Board and conducted in accordance with the Canadian Council on Animal Care.

### Experimental Procedures

**Figure 1:**
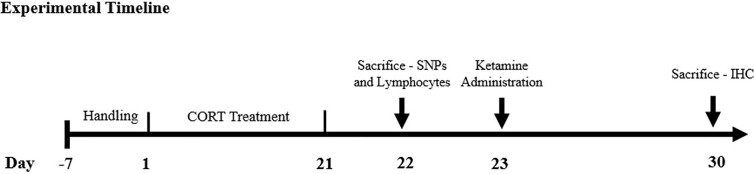
Experimental Timeline. The methodological timeline of the study. Rats were on a 12-h light-dark cycle that changed at 7am/pm and were injected between the h of 9am–10am to control for natural fluctuations in corticosterone (CORT) throughout the day. Sacrifices were conducted between the h of 9am–12pm. SNPs, synaptosomes; IHC, immunohistochemistry.

For a full experimental calendar, please see **Figure 1**. Handling of all rats took place for 7 consecutive days to habituate the animals to the procedure room and researcher before the first injection day. Animals in the CORT-21/Vehicle (Veh) and CORT-21/Ketamine group received subcutaneous injections of CORT dissolved in saline with 2% tween for 21 days. This is reliable and robust model for the study of depression that is able to be reversed with antidepressants (reviewed in Sterner and Kalynchuk, 2010). Animals in the CORT-21/Vehicle (Veh) and Animals in the Veh/Veh and Veh/Ket (ketamine) group received subcutaneous injections of saline with 2% Tween-80 for 21 days. For animals in the Veh/Ket and CORT-21/Ket group, ketamine hydrochloride (Vetalar; Bioniche Animal Health Canada Inc.; Belleville, Ontario, Canada) was suspended in saline and injected intraperitoneally on day 22 at a dose of 15 mg/kg, and a volume of 1 ml/kg.

Animals received subcutaneous injections of saline or of CORT for 21 consecutive days. CORT (Steraloids Inc.; Newport, Rhode Island, United States) was suspended in saline with 2% Tween80 (VWR International; West Chester, Pennsylvania, United States) at a dose of 40mg/kg and a volume of 1 ml/kg. This dose and injection paradigm were selected based on previous findings that 21 days of 40 mg/kg CORT produces reliable increases in depression-like behavior in rodents (Kalynchuk et al., 2004). Ketamine hydrochloride was suspended in saline and injected intraperitoneally at a dose of 15 mg/kg, and a volume of 1 ml/kg to a subset of animals.

### Immunohistochemistry

Animals (N=32) were anesthetized with 5% isofluorane and perfused with 4% paraformaldehyde, in which their brains were kept in the same fixative for 48 h at 4^°^C. They were then sectioned in the coronal plane at 30 µm on a cryostat (CM1850 UV, Leica Biosystems) at −20˚C. Sections were stored in cryoprotectant [30% (w/v) sucrose, 1% (w/v) polyvinylpyrrolidone, and 30% (v/v) ethylene glycol in 0.1 M PBS (pH = 7.4)] until use. Sections were rinsed in tris-buffered saline (TBS) (50 mM Tris-Cl, 150 mM NaCl; pH 7.6) and incubated in sodium citrate (pH 6; 85 °). After, the sections were incubated at room temperature for 24 h with rabbit anti-doublecortin (DCX) primary antibody or mouse anti-reelin antibody (1:1,000, EMD Millipore, Burlington, MA) diluted in a blocking solution consisting of 10% Triton X-100 [*v/v*], 15% normal goat serum (NGS) [*w/v*], and 1% bovine serum albumin (BSA) [*w/v*] dissolved in TBS. After primary antibody incubation, the sections were incubated in 10% hydrogen peroxide [*v/v*] in TBS for 30 min. The tissue was then incubated with biotinylated horse anti-mouse lgG (1:500, Sigma-Aldrich, St. Louis, MO) or biotinylated goat anti-rabbit lgG (1:500; Sigma-Aldrich, St. Louis, MO) secondary antibody diluted in the blocking solution described above for 1 h at room temperature. Each step was followed with rinses in TBS. Next, tissue was incubated in an avidin-biotin complex (1:500, Vecta Stain Elite ABC reagent, Vector Labs) for 1 h at room temperature. For reelin, sections were stained using 0.002% [*w/v*], 3’-diaminobenzidine (DAB, Sigma-Aldrich, St. Louis, MO) dissolved in 0.0078% [*v/v*] H_2_O_2_ in TBS. For DCX, slices were then washed 2 times in TBS and 1 time in sodium acetate before being stained using 0.025% DAB [*w/v*] and 4.167% NiSO4 [*w/v*] dissolved in 0.002% H_2_0_2_ and sodium acetate. The sections were mounted and coverslipped using Permount solution.

DCX was used as a marker to quantify and categorize cells from the proliferative stages of neurogenesis until that of an immature neuron, as it has a high level of specificity for these cells in the subgranular and granular zone of the hippocampus, and allows for categorization throughout the maturation process.

### Imaging

Sections were imaged using a Nikon Eclipse E800 microscope. Positive cells in the subgranular zone were counted and categorized (for DCX) using an unbiased optical fractionator method in Stereo Investigator (Version 8.0, MicroBrightField Inc.). These areas were traced at 4x magnification, and stereological analyses were undertaken at 40x magnification, with a field size of 3600 µm^2^, and number estimates were calculated through the following formula: Ntotal: ΣQ- × 1/ssf × A(x,y step)/a(frame) × t/h; where ΣQ- is the number of counted cells; ssf is the section sampling fraction (1/6); A is the area associated with each x,y movement (10,000 μm^2^); a(frame) is the area of the counting frame (3,600 μm^2^); t is the weighted average section thickness; and h is the height of the dissector P plane. A guard zone of 2 μm was used to prevent sectioning artifacts. DCX-positive cells were categorized using a method previously described by Plumpe et al. (2006).

### Synaptosomes

Control (N=4) and CORT (N=4) animals were anesthetized with 5% isofluorane and killed by decapitation. Immediately after sacrifice, the hippocampus and cerebellum were dissected on ice and snap frozen in liquid nitrogen then stored until use at −80˚C.

Tissue was thawed on ice prior to homogenization in ice using a Potter-Elvehjem homogenized containing chilled modified Krebs-Henseleit buffer (mKRBS) (in mM: 118.5 NaCl, 4.70 KCl, 1.18 MgCl2·6H2O, 2.50 CaCl2·2H2O, 1.18 KH2PO4, 24.90 NaHCO3, 10.00 glucose, pH adjusted to ~7.40 using 1.0 N HCl) supplemented with a protease inhibitor cocktail (#1860932, Thermoscientific, Waltham, MA). A small portion of this whole homogenate was stored for later analysis. As previously described the remaining homogenate was used to prepare a subfraction containing intact synaptosomes (SN) (Brymer et al., 2020). After centrifugation, the SN pellet was resuspended in pre-warmed artificial cerebrospinal fluid (ACSF) (32˚C) and divided evenly between drug experimental groups. The hippocampal SNs were then incubated for 30 min at 32˚C with 5% circulating CO_2_ with either reelin at a concentration of 5 nM, 10 nM, or 50 nM, ketamine at a concentration of 50 nM, 100 nM, 500 nM, or ACSF alone. These concentrations were based off previous research from our lab and others (Li et al., 2010). Cerebellar SNs followed an identical protocol, except they were incubated with 5 nм, 10 nм, or 50 nм of ketamine. Reactions were terminated with a high centrifugation (3,490 x g for 15 min) at 4˚C in ice-cold mKRBS. Pellets were resuspended in 50 μl mKRBS supplemented with protease and phosphatase inhibitor cocktails (78428, Thermoscientific, Waltham, MA). 15 μl of this homogenate was used for detergent compatible (DC) protein assay analysis (#5000111, BioRad, Hercules, CA).

### Western Blotting

For Western blot analysis, 10 μg of protein was electrophoretically resolved in 10% SDS-polyacrylamide gel at 200 V for 60 min, then transferred onto polyvinylidene fluoride membranes (#IPVH00010, Millipore Sigma, Burlington, MA) *via* wet transfer (100 V on ice for 90 min). Membranes were blocked using 5% (w/v) BSA for 1 h at room temperature. For all groups of SNs, PSD-95, total mTOR (#2983T, Cell Signaling Technology, Danvers, MA), and phosphorylated mTOR (p-mTOR) (#2971S, Cell Signaling Technology, Danvers, MA) were measured. All antibodies were diluted in 10 ml of the 5% [*w/*v] BSA blocking buffer and applied to the blots overnight at 4˚C with gentle agitation. Blots were washed in tris-buffered saline with 1% [*v/*v] tween following incubation, and then incubated with horse radish peroxidase linked goat anti-mouse or goat anti-rabbit secondary antibody diluted at 1:5,000 in blocking buffer for 1 h at room temperature. Luminata Crescendo (for p-mTOR and mTOR) or Classico (PSD-95) (#WBLUR0500 and # WBLUC0500, Millipore Sigma, Burlington, MA) were used for chemiluminescent detection. All images were captured using a SynGene imaging system. Western blot bands were quantified using GeneTools companion program. Ponceau staining was used as the normalization standard, as it is more sensitive to differences in loading amounts than standard housekeeping proteins (Thacker et al., 2016).

### Lymphocyte Isolation and Incubation

Lymphocytes were extracted following a previous protocol established in our lab from controls (Romay-Tallon et al., 2018). These extracted lymphocytes were then resuspended in RPMI 1640 medium (#11875093, Thermoscientific, Waltham, MA) diluted with 10% phosphate-buffered saline (PBS) (137 mM NaCl, 2.7 mM KCl, 10 mM Na_2_HPO_4_, 1.8 mM KH_2_PO_4_ ; pH 7.4) [*w/v*] and 1% streptomycin and incubated first with either medium or 1 mM CORT, and then with varying concentrations of reelin (0.5 nM, 1 nM, 5 nM) and ketamine (10 nM, 50 nM, 100 nM, 250 nM) in the medium specified above. 1% [*w/v*] paraformaldehyde was added for 5 min to the solution to fix the lymphocytes, and then was centrifuged in 1:1 PBS (two times, 1,000 x g, 10 min) to rinse. 1 mM of CORT was used as it is equivalent to the 40mg/kg injected in our rat model and has paralleled changes that we have observed in patients with depression (Romay-Tallon et al., 2018).

After incubation, lymphocytes were incubated for 10 min at 4°C in a 100 ml solution of 3% rat immunoglobulin G (IgG) diluted in PBS with 1% BSA [*w/v*] (PBS + BSA), then for 24 h at 4°C in rabbit anti-serotonin transporter (SERT) antibody (#AB9322, Millipore Sigma, Burlington, MA), diluted 1:100 in PBS+BSA. After washing 3x in PBS for 10 min, samples were incubated with goat anti-rabbit secondary antibody (#ab175471, abcam, Cambridge, UK) diluted 1:250 in PBS+BSA and conjugated with Alexa Fluor 568 in the dark for 1 h at room temperature and then for 10 min in Hoechst diluted 1:1,000 in PBS. After 3× 10 min washes, samples were extended onto slides and cover-slipped with Citifluor-Mount Solution (Electron Microscopy Science) and stored at −20°C until analysis. Through compound microscopy, lymphocytes were analyzed using ImageJ to quantify the number and size of SERT clusters.

### Statistics

Immunohistochemistry data was analyzed using two-tailed independent t-tests. Incubations of SNs were grouped together and compared against control and CORT subgroups using a Welch’s corrected t-test to account for uneven standard deviations. Individual incubations were compared against vehicle and CORT subgroups using two-tailed independent t-tests to determine individual impacts. SERT cluster number and size on lymphocytes were examined identically. Data are represented as the mean ± SEM or SD.

## Results

### Ketamine Rescues Reelin Expression in the Dorsal Dentate Gyrus SGZ

**Figure 2:**
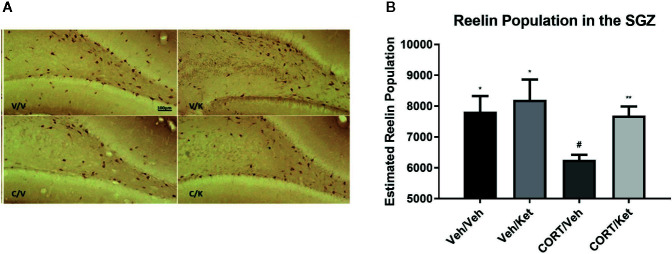
The effect of corticosterone (CORT) and ketamine on reelin-IR cells in the gyrus subgranular zone (SGZ). **(A)** Representative photomicrographs of reelin-ir cells in the dorsal SGZ. Stereological analyses were undertaken at 40× magnification. **(B)** Effects of treatment on reelin-ir cells in the dorsal SGZ. CORT significantly decreased the number of positive cells, which was rescued by acute ketamine administration. There were no effects of CORT or reelin in the ventral hippocampus. All data are expressed as mean±SD. Veh/Veh, vehicle/vehicle; Veh/Ket, vehicle/ketamine; CORT/Veh, CORT/vehicle; CORT/Ket, CORT/ketamine. *p < 0.05 vs. Veh/Veh, ^#^p < 0.05, **p < 0.01 vs. CORT/Veh.

Analyses of the dorsal SGZ showed CORT administration decreased reelin immunoreactive cells (p = 0.0198, t = 2.902, df = 8), which was rescued by ketamine administration (p = 0.0034, t = 3.932, df = 9) (**Figures 2A, B**).

### Doublecortin Counts and Categorization in the SGZ

**Figure 3:**
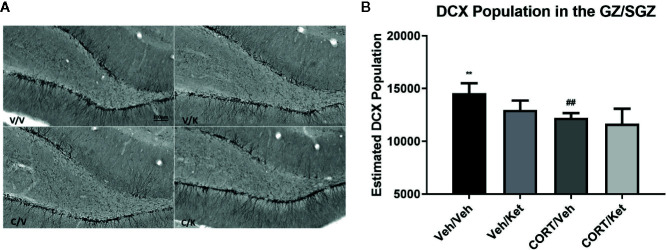
The effect of corticosterone (CORT) and ketamine on doublecortin (DCX)-IR cells in the gyrus subgranular zone (SGZ). **(A)** Representative photomicrographs of DCX-ir cells in the SGZ. **(B)** Effects of treatment on DCX-ir cells. CORT significantly decreased the number of positive cells, which was not rescued with acute ketamine administration. All data are expressed as mean±SD. Veh/Veh, vehicle/vehicle; Veh/Ket, vehicle/ketamine; CORT/Veh, CORT/vehicle; CORT/Ket, CORT/ketamine. **p < 0.01 vs. Veh/Veh, ^##^p < 0.01 vs. CORT/Veh.

Doublecortin was used as a marker for neural maturation of newborn granule cells in the subgranular zone of the hippocampus, as it allows for both cell-counts and categorization of dendritic branching. Histological data showed large gaps of doublecortin positive cells in the upper blade of the dentate gyrus after CORT administration, an effect that was not rescued through ketamine administration (**Figure 3**). Significant differences were found between the controls and CORT-treated animals (p = 0.0057, t = 3.735, df = 8), but not any other subgroups, suggesting ketamine did not influence doublecortin levels in this sample. No significant correlations were found between subgroups and complexity of dendritic branching on the newborn granule cells.

### Hippocampal Synaptosomes

**Figure 4:**
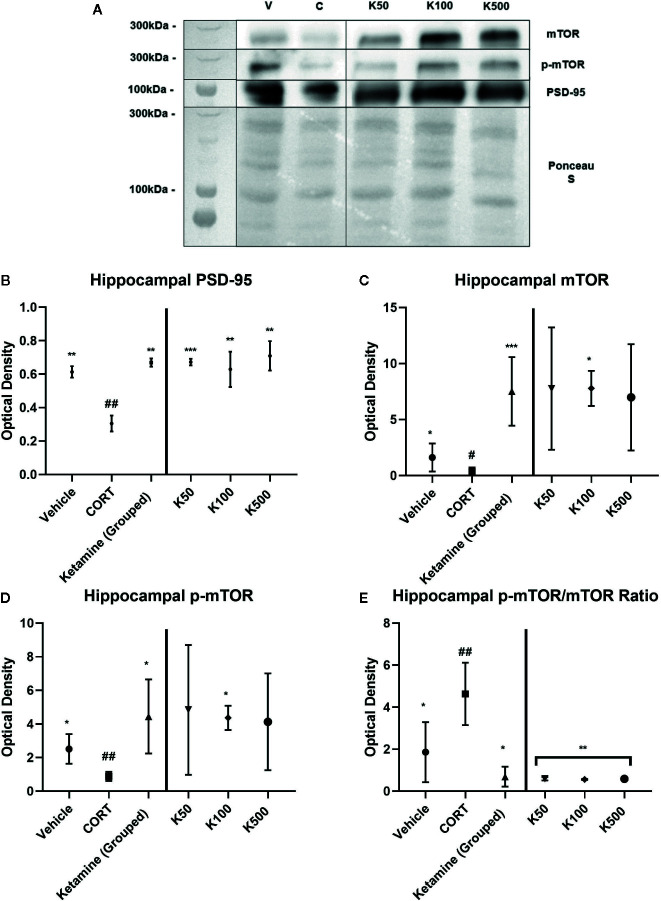
The effect of ketamine on synaptic-level proteins in hippocampal synaptosomes. **(A)** Representative Western Blot images of synaptic-level protein expression in the hippocampus after ketamine incubations on synaptosomes derived from corticosterone (CORT)-treated animals. Ladder presented is Spectra Multicolor High Range Protein Ladder (Thermofisher, #26625). **(B)** Effects of ketamine incubations on postsynaptic density-95 (PSD-95). CORT decreased this expression while all ketamine incubations increased these levels. **(C)** Effects of ketamine incubations on mammalian target of rapamycin (mTOR). All concentrations of ketamine increased mTOR levels above CORT-treated animals. **(D)** Effects of ketamine incubations compared to those of reelin on p-mTOR. Only 10nм of ketamine increased p-mTOR levels above CORT-treated animals. **(E)** Effects of ketamine incubations on the active ratio of mTOR. All data are expressed as mean±SD. K50, 50nм ketamine; K100, 100nм ketamine; K500, 500nм ketamine. ^#^p < 0.05, ^##^p < 0.01 vs. vehicle, *p < 0.05, **p < 0.01, ***p < 0.001 vs. CORT.

The impact of ketamine incubations on SN were used to determine similarities with our previous research that showed reelin increases the protein expression levels of PSD-95, mTOR, and p-mTOR (see **Figures 4A–E** for comparative data). The incubations of hippocampal SNs from CORT-treated animals with ketamine were run in parallel to those of reelin published previously (Brymer et al., 2020), and thus have equivalent data-points for the vehicle and CORT experimental subgroups. Grouped concentrations of ketamine increased PSD-95 expression from CORT-administered animals (p = 0.0012, t = 6.803, df = 4.78) (**Figure 4B**), as well as each individual concentration (50 nM, p = 0.0005, t = 6.721, df = 6; 100 nM, p = 0.0026, t = 4.931, df = 6; 500 nM, p = 0.0015, t = 5.539, df = 6). Similar differences were found in grouped mTOR expression (p=0.0001, t = 5.452, df = 12.8) (**Figure 4C**), though only one concentration of ketamine significantly increased levels above CORT-administered animals (100 nM, p = 0.0165, t = 3.296, df = 6) due to high variability in the other concentration groups. Though p-mTOR was also only significant at 100nM (p = 0.0165, t = 3.296, df = 6) due to high variability (see **Figure 4D**), when treatment effects were grouped ketamine did significantly increase p-mTOR (p = 0.0103, t = 3.916, df = 5.22). Ketamine did not increase mTOR activity at any concentration, though it interestingly decreased it back to levels that were found in the control animals (**Figure 4E**).

### Cerebellar Synaptosomes 

**Figure 5:**
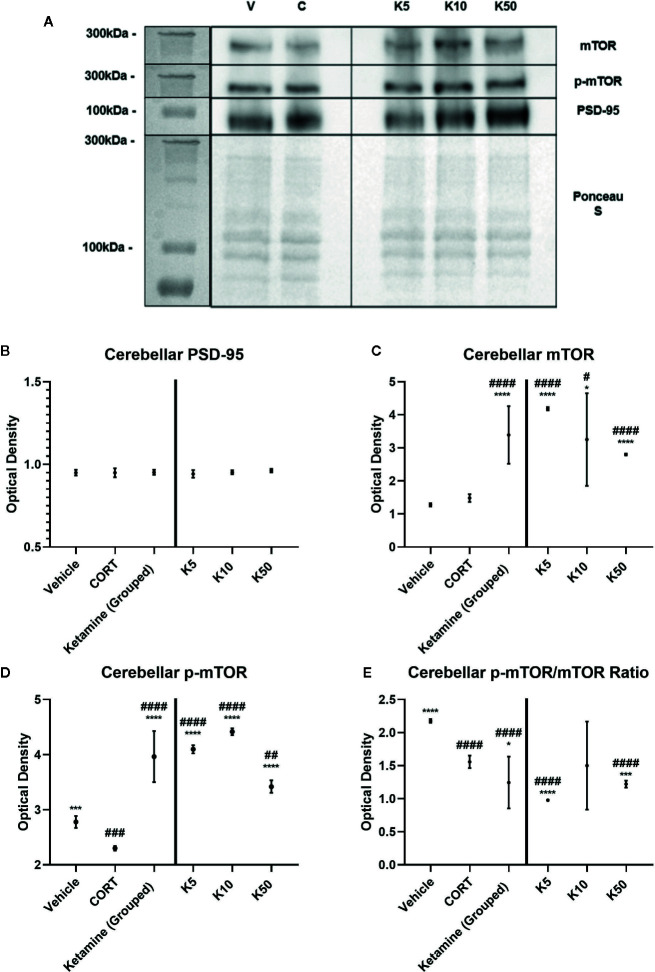
The effect of ketamine on synaptic-level proteins in cerebellar synaptosomes. **(A)** Representative Western Blot images of synaptic-level protein expression in the cerebellum after ketamine incubations on synaptosomes from corticosterone (CORT)-treated animals. Ladder presented is Spectra Multicolor High Range Protein Ladder (Thermofisher, #26625). **(B)** Effects of ketamine incubations on levels of postsynaptic density-95 (PSD-95). All levels stayed the same. **(C)** Effects of ketamine incubations on levels of mammalian target of rapamycin (mTOR). All concentrations of ketamine increased the levels above vehicle and CORT. **(D)** Effects of ketamine on levels of p-mTOR. All concentrations were significantly different from both vehicle and CORT. **(E)** Effects of ketamine on the active ratio of mTOR. All data are expressed as mean±SD. K5, 5nм ketamine; K10, 10nм ketamine; K50, 50nм ketamine. ^#^p < 0.05, ^##^p < 0.01, ^###^p < 0.001, ^####^p < 0.0001 vs. vehicle, *p < 0.05, ***p < 0.001, ****p < 0.0001 vs. CORT.

**Figure 6:**
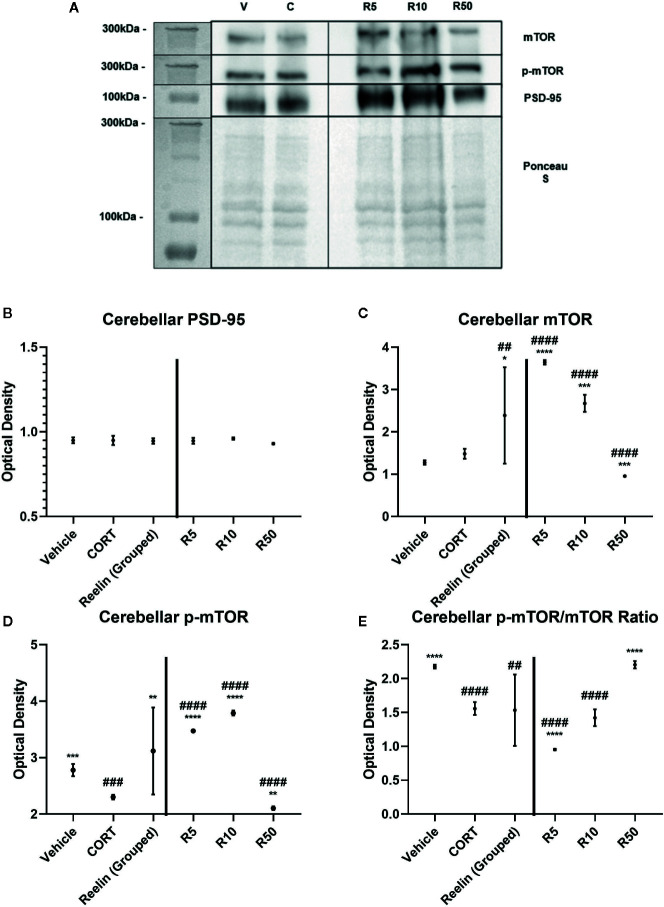
The effect of reelin on synaptic-level proteins in cerebellar synaptosomes. **(A)** Representative Western Blot images of synaptic-level protein expression in the cerebellum after reelin incubations on synaptosomes from corticosterone (CORT)-treated animals. Ladder presented is Spectra Multicolor High Range Protein Ladder (Thermofisher, #26625). **(B)** Effects of reelin incubations on levels of postsynaptic density-95 (PSD-95). All levels stayed the same. **(C)** Effects of reelin incubations on levels of mammalian target of rapamycin (mTOR). Lower concentrations increased the levels above vehicle and CORT, but higher concentrations decreased mTOR levels. **(D)** Effects of reelin on levels of p-mTOR. All concentrations were significantly different from both vehicle and CORT, paralleling the findings with mTOR. **(E)** Effects of ketamine and reelin on the active ratio of mTOR. All data are expressed as mean±SD. R5, 5nм reelin; R10, 10nм reelin; R50, 50nм reelin. ^##^p < 0.01, ^###^p < 0.001, ^####^p < 0.0001 vs. vehicle, *p < 0.05, **p < 0.01, ***p < 0.001, ****p < 0.0001 vs. CORT.

To ascertain whether the displayed synaptic-level effects of reelin and ketamine are restricted to the hippocampus, we analyzed the effects of reelin and ketamine in the cerebellum, an area with a high concentration of reelin. Representative Western Blot images are displayed in **Figures 5A** and **6A**. In the cerebellar SN there were no significant differences in PSD-95 expression between any subgroups (vehicle; CORT; reelin at 5 nM, 10 nM, 50 nM; ketamine at 5 nM, 10 nM, 50 nM), a sharp contrast to the previous findings in the hippocampus (**Figures 5B** and **6B**).

There were no differences found between vehicle and CORT in the total expression of mTOR. However with ketamine treatment, significant increases above CORT and vehicle levels were found with both grouped (against vehicle: p <0.0001, t = 8.358, df = 11.1) (against CORT: p<0.0001, t = 7.390, df = 12) and individual analyses (against CORT: 5 nM, p<0.0001, t = 20.5, df = 6; 10 nM, p = 0.0238, t = 3.007, df = 6, 50 nM, p<0.0001, t = 8.791, df = 6) (**Figure 5C**). This was paralleled after reelin incubations, having both combined (against CORT: p = 0.019, t = 2.722, df = 11.6) and individual (against CORT: 5 nM, p<0.0001, t = 25.32, df = 6; 10 nM, p = 0.0002, t = 8.395, df = 6; 50 nM, p = 0.0001, t = 8.791, df = 6) impact on mTOR expression (**Figure 6C**).

p-mTOR expression showed a similar pattern to total mTOR, but revealed that controls had significantly higher levels of p-mTOR than CORT-treated animals (p = 0.0002, t = 8.328, df = 6). Ketamine once again increased p-mTOR expression with both pooled (against CORT: p<0.0001, t = 12.28, df = 11.5) and individual (5 nM, p<0.0001, t = 56.57, df = 6; 10 nM, p<0.0001, t = 43.06, df = 6; 50 nM, p<0.0001, t = 11.27, df = 6) concentrations (**Figure 5D**). Reelin, while still having a pooled effect (p = 0.0037, t = 3.658, df = 11.1), was more effective in lower concentrations, with 5 nM (p<0.0001, t = 34.66, df = 6) and 10 nM (p<0.0001, t = 41.01, df = 6) more effectively increasing protein expression than 50 nM (p = 0.0013, t = 5.625, df = 6) (**Figure 6D**).

The ratio of activated mTOR was lower in CORT-treated animals than the vehicles (p<0.0001, t = 12.45, df = 6), an opposite effect of what occurred in the hippocampus. When pooled, ketamine treatment decreased this activity (p = 0.0234, t = 2.55, df = 1.6), particularly with 5 nM (p<0.0001, t = 11.03, df = 6) and 50 nM (p = 0.0005, t = 6.908, df = 6) incubations (**Figure 5E**). Grouped reelin treatment had no effect, as 5 nM (p<0.0001, t = 11.6, df = 6) significantly decreased the ratio from CORT, whereas 50 nM (p<0.0001, t = 12.47, df = 6) increased it (**Figure 6E**).

### SERT Labeling in Lymphocytes 

**Figure 7:**
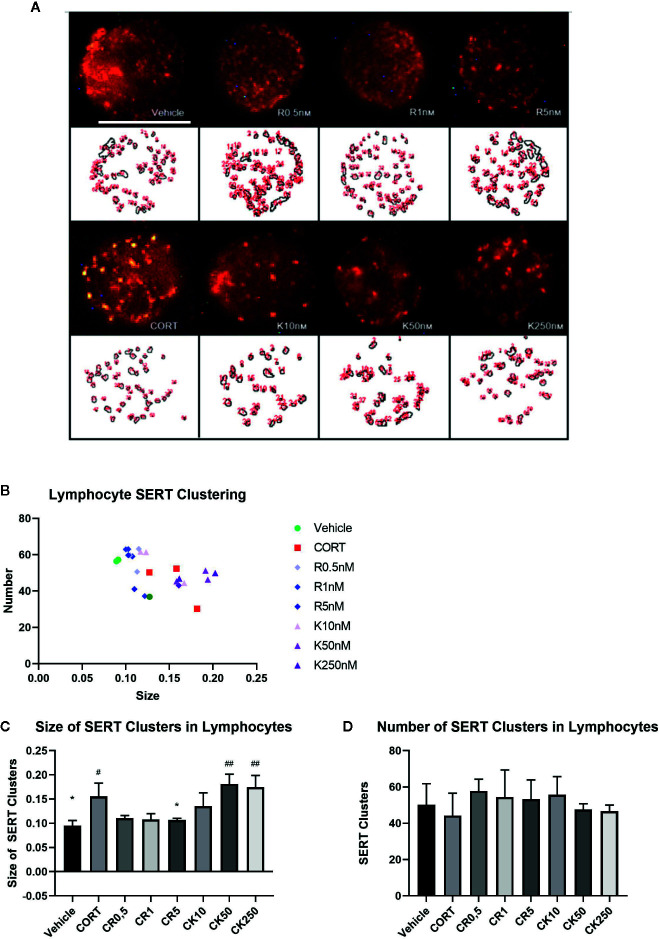
The effect of corticosterone (CORT), reelin, and ketamine on serotonin transporter (SERT) clusters in peripheral lymphocytes. **(A)** Representative confocal microscopy images for each condition. **(B)** Effect of treatment on clusters by number and size. **(C)** Effect of treatment on size of SERT clusters. CORT increased the size of SERT clusters, which was rescued by reelin but not ketamine. **(D)** Effect of treatment on number of SERT clusters. No changes were found between any groups. All data expressed as mean±SD. ^#^p < 0.05 vs. vehicle, *p < 0.05, ^##^p < 0.01 vs. CORT.

In both depression patients and the repeated CORT paradigm our lab has found parallel changes in the analysis of serotonin transporter (SERT) clustering in the plasma membranes of lymphocytes (Romay-Tallon et al., 2018), and thereby we wanted to ascertain the effects of ketamine and reelin on SERT clustering. **Figure 7** shows representative images of lymphocytes from vehicle, CORT, reelin, and ketamine treated lymphocytes at varying concentrations. There was a significant difference in size, but not number, of SERT clusters in all experimental groups. As expected, CORT treatment increased the size of SERT clusters from the vehicle (p = 0.0236, t = 3.56, df = 4). Only the highest concentration reelin decreased the size of clusters (5 nM, p = 0.0375, t = 5.278, df = 4) shifting them back to the patterns of the vehicle. At the lowest dose (10 nM) ketamine had a similar effect to reelin though it was not significant. Higher doses of ketamine appeared to increase the size of clusters from those of CORT-treated animals, though this was not significant. There was no significant impact on number of clusters in any experimental groups.

## Discussion

In the present report we provided evidence that ketamine is able to rescue the deficits of reelin but not DCX induced by repeated CORT in the dentate gyrus subgranular zone. We also demonstrated that both ketamine and reelin are able to rescue the deficits in mTOR and p-mTOR induced by CORT in both hippocampal and cerebellar synaptosomes, and that reelin, but not ketamine, corrects the alterations in SERT clustering in lymphocytes induced by CORT.

We used a well-defined model with daily injections of CORT for three weeks that consistently results in heavy alterations in immobility in the forced swim test, a paradigm widely used to evaluate antidepressant drug activity (Yuen et al., 2017).We have previously shown that repeated-CORT results in a significant decrease in reelin expression in the dentate gyrus subgranular zone which could have a possible effect on newborn cell maturation and on regulating synaptic strength of synapses impinging onto the dendritic spines of granule cells, particularly in the distal dentate molecular layer and the *stratum lacunosum-moleculare*. These connections could underlie some of the depressive-like behaviors observed in these animals (Lussier et al., 2009; Lussier et al., 2011; Lussier et al., 2013). Deficits in reelin expression in the dentate SGZ are rescued by both conventional (e.g. imipramine) and non-conventional (e.g. etanercept) drugs with antidepressant actions, which also reverse the increase in immobility in the forced swim test induced by CORT (Fenton et al., 2015; Brymer et al., 2018). Considering this, we expected ketamine to also be able to reverse the decrease in reelin+ cells in the SGZ in a fast-acting manner, which is consistent with our results. Recently, we demonstrated that intrahippocampal infusions of recombinant reelin are able to also rescue the behavioral phenotype induced by repeated CORT and that they do so in a fast-acting manner (i.e. similar to ketamine) (Brymer et al., 2020). This brings about the possibility that rescuing hippocampal reelin may parallel ketamine’s actions in its fast-acting antidepressant effects, similar to other potential fast-acting antidepressant molecules that have been studied (Pazini et al., 2016; Hasegawa et al., 2019; de Almeida et al., 2020). Further research should be conducted to determine if ketamine’s antidepressant effects are augmented by the increase of reelin expression it stimulates in the hippocampus.

CORT had a significant impact on hippocampal neurogenesis, decreasing the populations of newborn and immature neurons in the subgranular zone. These data are supported by numerous other reports, which show consistent and significant decreases in hippocampal cell proliferation and survival in the adult rodent brain (Pham et al., 2003; Wong and Herbert, 2006; Brummelte and Galea, 2010). The administration of an acute dose of ketamine did not rescue adult neurogenesis in the chronic stress animal model in the same way that a single infusion of Reelin did not rescue DCX expression while repeated reelin infusions did (Brymer et al., 2020). However, mixed results regarding proliferation and maturation after a single dose of ketamine suggest that increased neurogenesis is not sufficient for the short term behavioral and biological antidepressant effects following acute administration (Doherty et al., 2016; Soumier et al., 2016), but rather is potentially associated with the long-term antidepressant effects found after chronic ketamine administration (Clarke et al., 2017; Ma et al., 2017). Thereby, it seems that both acute ketamine and reelin do not have a fast effect in rescuing the effects on hippocampal neurogenesis (as ascertained by DCX labeling) but do so when administered repeatedly. It is therefore a possibility that the acute effects of reelin and ketamine would have a major incidence in the reversal of some depressive symptoms, while chronic effects might be more effective in rescuing other symptoms (i.e. depression cognitive deficits), but further experiments are necessary to elucidate this. Research in the ventral hippocampus would also be of interest, as it has been shown that the dorsal and ventral hippocampus are differentially affected by glucocorticoid administration and could have been disparately impacted by ketamine (Levone et al., 2020). It is important to note that researchers have recently demonstrated that DCX expression can increase in the absence of adult hippocampal neurogenesis, implicating that DCX does not always indicate the presence of neurogenesis (Mendez-David et al., 2020), so caution should be used when interpreting these results.

To gain insight into possible common molecular mechanisms of ketamine and reelin, we incubated hippocampal and cerebellar synaptosomes obtained from vehicle and CORT treated animals with different concentrations of ketamine or reelin. This experiment showed that CORT decreased levels of PSD-95 expression in hippocampal synaptosomes that were rescued both by ketamine and reelin in a concentration dependent manner (see Brymer et al., 2020), but not in cerebellar synaptosomes where CORT, ketamine, and reelin did not have an effect on PSD-95 levels. This could indicate perhaps a more stable nature of cerebellar synapses in adulthood in comparison to those in the hippocampus, which are more sensitive to plastic and stress-induced changes. However, while there is a general decrease in mTOR and p-mTOR expression in both hippocampal and cerebellar synaptosomes that is rescued by both ketamine and reelin, it seems that the effects of ketamine are larger than those of reelin. This might be of interest when considering that while mTOR activation has been considered a putative molecular mechanism underlying the fast antidepressant actions of ketamine (Zhou et al., 2014; Zanos et al., 2016; Zanos et al., 2018), a recent report has surprisingly shown that rapamycin (a mTOR inhibitor) prolonged the antidepressant effects of ketamine (Abdallah et al., 2020). This suggests that a more moderate activation of mTOR (i.e. like the one induced by reelin) could be equally effective, and would perhaps avoid the psychotomimetic effects of ketamine (Krystal et al., 1994), that necessitate hospital administration and make it a undesirable treatment for varied patient populations, such as those with comorbid schizophrenia. Although this is quite speculative at the moment, it would be of interest to evaluate considering that levels of reelin levels in the brains of schizophrenia patients are nearly 50% downregulated in comparison to controls. (Impagnatiello et al., 1998; Fatemi et al., 2000; Guidotti et al., 2000).

The importance of investigating potential peripheral mechasims of depression and therapeutics led us to investigate SERT clustering on peripheral lymphocytes, which appears to be influenced by reelin expression as shown in our lab previously (Rivera-Baltanas et al., 2010; Rivera-Baltanas et al., 2012). The quantification of SERT clusters labeling in the membrane of peripheral lymphocytes resulted in an increase of SERT-clusters size induced by CORT, as it was previously shown by Romay-Tallon et al. (2018), that was rescued by incubation with recombinant reelin for 30 min but not by ketamine. This suggests that reelin may have a peripheral effect on lymphocytes that is not observed in ketamine, and could be an idea worth pursuing (i.e. the peripheral effects of reelin) when considering that similar alterations to those induced by CORT in SERT-clusters have been observed in naive depression patients, and were considered to represent a putative biomarker of therapeutic efficacy of antidepressant medication (Rivera-Baltanas et al., 2012; Rivera-Baltanas et al., 2015; Caruncho et al., 2019).

It is important to note certain limitations in this study. A small number of samples limits statistical power in each experiment. As previously discussed, DCX is not necessarily a reliable indicator for adult hippocampal neurogenesis. This was a pilot study to determine ketamine’s impact on reelin expression, as well as any potential parallels between ketamine and reelin’s effects on protein expression in the synapse, as well as in peripheral lymphocytes. However, the inclusion of only adult males limits the extrapolation of results. The inclusion of females is an essential next step, as differing manifestations of depression and treatment responsiveness between sexes necessitate further investigation into unique cellular mechanisms (recently reviewed in Eid et al., 2019).

In conclusion, our research has shown that ketamine brings about a fast-rescuing effect of reelin expression in the hippocampal subgranular zone, and that reelin may perhaps work through similar synaptic mechanisms to ketamine that perhaps underlie the fast-acting antidepressant effects of this drug. However, reelin shows some peripheral effects in SERT-clustering that are not shown by ketamine. It would be of interest to further evaluate the ketamine/reelin pathways in both CNS and periphery to not only gain additional insight on ketamine actions but also to determine the potential fast antidepressant-like actions of reelin.

## Data Availability Statement

The original contributions presented in the study are included in the article/supplementary material; further inquiries can be directed to the corresponding author.

## Ethics Statement

The animal study was reviewed and approved by Ethics board University of Saskatchewan; Ethics board University of Victoria.

## Author Contributions

JJ, LK, and HC designed the study and wrote the manuscript. JJ, JT, CD, BK, and RR-T obtained the data and reviewed the manuscript.

## Funding

Supported by NSERC Discovery Grants to LK and HC. JJ was supported by a NSERC MSc scholarship.

## Conflict of Interest

The authors declare that the research was conducted in the absence of any commercial or financial relationships that could be construed as a potential conflict of interest.

## References

[B1] Abdallah C. G. Averill L. A. Gueorguieva R. Goktas S. Purohit P. Ranganathan M. (2020). Modulation of the antidepressant effects of ketamine by the mTORC1 inhibitor rapamycin. Neuropsychopharmacology. 10.1038/s41386-020-0644-9. PMC7162891 32092760

[B2] Beffert U. Weeber E. J. Durudas A. Qiu S. Masiulis I. Sweatt J. D. (2005). Modulation of synaptic plasticity and memory by Reelin involves differential splicing of the lipoprotein receptor Apoer2. Neuron 47, 567–579. 10.1016/j.neuron.2005.07.007 16102539

[B3] Bosch C. Masachs N. Exposito-Alonso D. Martínez A. Teixeira C. M. Fernaud I. (2016). Reelin Regulates the Maturation of Dendritic Spines, Synaptogenesis and Glial Ensheathment of Newborn Granule Cells. Cereb. Cortex 26, 4282–4298. 10.1093/cercor/bhw216 27624722 PMC5066826

[B4] Brummelte S. Galea L. A. M. (2010). Chronic corticosterone during pregnancy and postpartum affects maternal care, cell proliferation and depressive-like behavior in the dam. Horm. Behav. 58, 769–779. 10.1016/j.yhbeh.2010.07.012 20688070

[B5] Brymer K. J. Fenton E. Y. Kalynchuk L. E. Caruncho H. J. (2018). Peripheral Etanercept Administration Normalizes Behavior, Hippocampal Neurogenesis, and Hippocampal Reelin and GABAA Receptor Expression in a Preclinical Model of Depression. Front. Pharmacol. 9, 121. 10.3389/fphar.2018.00121 29515447 PMC5826281

[B6] Brymer K. J. Johnston J. Botterill J. J. Romay-Tallon R. Mitchell M. A. Allen J. (2020). Fast-acting antidepressant-like effects of Reelin evaluated in the repeated-corticosterone chronic stress paradigm. Neuropsychopharmacology 45, 1707–1716. 10.1038/s41386-020-0609-z 31926481 PMC7419539

[B7] Caruncho H. J. Rivera-Baltanas T. Romay-Tallon R. Kalynchuk L. E. Olivares J. M. (2019). Patterns of Membrane Protein Clustering in Peripheral Lymphocytes as Predictors of Therapeutic Outcomes in Major Depressive Disorder. Front. Pharmacol. 10, 190. 10.3389/fphar.2019.00190 30930773 PMC6423346

[B8] Chameau P. Inta D. Vitalis T. Monyer H. Wadman W. J. van Hooft J. A. (2009). The N-terminal region of reelin regulates postnatal dendritic maturation of cortical pyramidal neurons. Proc. Natl. Acad. Sci. U. S. A. 106, 7227–7232. 10.1073/pnas.0810764106 19366679 PMC2678467

[B9] Clarke M. Razmjou S. Prowse N. Dwyer Z. Litteljohn D. Pentz R. (2017). Ketamine modulates hippocampal neurogenesis and pro-inflammatory cytokines but not stressor induced neurochemical changes. Neuropharmacology 112, 210–220. 10.1016/j.neuropharm.2016.04.021 27106168

[B10] Dantzer R. O’Connor J. C. Freund G. G. Johnson R. W. Kelley K. W. (2008). From inflammation to sickness and depression: when the immune system subjugates the brain. Nat. Rev. Neurosci. 9, 46–56. 10.1038/nrn2297 18073775 PMC2919277

[B11] de Almeida R. F. Pocharski C. B. Rodrigues A. L. S. Elisabetsky E. Souza D. O. (2020). Guanosine fast onset antidepressant-like effects in the olfactory bulbectomy mice model. Sci. Rep. 10, 8429. 10.1038/s41598-020-65300-w 32439951 PMC7242421

[B12] Doherty F. King M. Wigmore P. Fone K. (2016). The effects of ketamine on behaviour, plasma corticosterone and neurogenesis in socially isolated rats. Eur. Neuropsychopharmacol. 26, S378. 10.1016/S0924-977X(16)31326-8

[B13] Eid R. S. Gobinath A. R. Galea L. A. M. (2019). Sex differences in depression: Insights from clinical and preclinical studies. Prog. Neurobiol. 176, 86–102. 10.1016/j.pneurobio.2019.01.006 30721749

[B14] Fatemi S. H. Earle J. A. McMenomy T. (2000). Reduction in Reelin immunoreactivity in hippocampus of subjects with schizophrenia, bipolar disorder and major depression. Mol. Psychiatry 5 (571), 654–663. 10.1038/sj.mp.4000783 11126396

[B15] Fenton E. Y. Fournier N. M. Lussier A. L. Romay-Tallon R. Caruncho H. J. Kalynchuk L. E. (2015). Imipramine protects against the deleterious effects of chronic corticosterone on depression-like behavior, hippocampal reelin expression, and neuronal maturation. Prog. Neuropsychopharmacol. Biol. Psychiatry 60, 52–59. 10.1016/j.pnpbp.2015.02.001 25681757

[B16] Friedrich M. J. (2017). Depression Is the Leading Cause of Disability Around the World. JAMA 317, 1517. 10.1001/jama.2017.3826 28418490

[B17] Guidotti A. Auta J. Davis J. M. Di-Giorgi-Gerevini V. Dwivedi Y. Grayson D. R. (2000). Decrease in reelin and glutamic acid decarboxylase67 (GAD67) expression in schizophrenia and bipolar disorder: a postmortem brain study. Arch. Gen. Psychiatry 57, 1061–1069. 10.1001/archpsyc.57.11.1061 11074872

[B18] Hasegawa Y. Zhu X. Kamiya A. (2019). NV-5138 as a fast-acting antidepressant via direct activation of mTORC1 signaling. J. Clin. Invest. 129, 2207–2209. 10.1172/JCI129702 31107245 PMC6546442

[B19] Hethorn W. R. Ciarlone S. L. Filonova I. Rogers J. T. Aguirre D. Ramirez R. A. (2015). Reelin supplementation recovers synaptic plasticity and cognitive deficits in a mouse model for Angelman syndrome. Eur. J. Neurosci. 41, 1372–1380. 10.1111/ejn.12893 25864922 PMC4676289

[B20] Ibrahim L. Diazgranados N. Luckenbaugh D. A. Machado-Vieira R. Baumann J. Mallinger A. G. (2011). Rapid decrease in depressive symptoms with an N-methyl-d-aspartate antagonist in ECT-resistant major depression. Prog. Neuropsychopharmacol. Biol. Psychiatry 35, 1155–1159. 10.1016/j.pnpbp.2011.03.019 21466832 PMC3100439

[B21] Impagnatiello F. Guidotti A. R. Pesold C. Dwivedi Y. Caruncho H. Pisu M. G. (1998). A decrease of reelin expression as a putative vulnerability factor in schizophrenia. Proc. Natl. Acad. Sci. U. S. A. 95, 15718–15723. 10.1073/pnas.95.26.15718 9861036 PMC28110

[B22] Kalynchuk L. E. Gregus A. Boudreau D. Perrot-Sinal T. S. (2004). Corticosterone increases depression-like behavior, with some effects on predator odor-induced defensive behavior, in male and female rats. Behav. Neurosci. 118, 1365. 15598145 10.1037/0735-7044.118.6.1365

[B23] Kelly J. Kennedy P. Cryan J. Dinan T. Clarke G. Hyland N. (2015). Breaking Down the Barriers: The Gut Microbiome, Intestinal Permeability and Stress-related Psychiatric Disorders. Front. Cell. Neurosci. 9, 392. 10.3389/fncel.2015.00392 26528128 PMC4604320

[B24] Kohler S. Cierpinsky K. Kronenberg G. Adli M. (2016). The serotonergic system in the neurobiology of depression: Relevance for novel antidepressants. J. Psychopharmacol. 30, 13–22. 10.1177/0269881115609072 26464458

[B25] Krystal J. H. Karper L. P. Seibyl J. P. Freeman G. K. Delaney R. Bremner J. D. (1994). Subanesthetic effects of the noncompetitive NMDA antagonist, ketamine, in humans. Psychotomimetic, perceptual, cognitive, and neuroendocrine responses. Arch. Gen. Psychiatry 51, 199–214. 10.1001/archpsyc.1994.03950030035004 8122957

[B26] Leonard B. E. (2018). Inflammation and depression: a causal or coincidental link to the pathophysiology? Acta Neuropsychiatr. 30, 1–16. 10.1017/neu.2016.69 28112061

[B27] Levone B. R. Codagnone M. G. Moloney G. M. Nolan Y. M. Cryan J. F. O’ Leary O. F. (2020). Adult-born neurons from the dorsal, intermediate, and ventral regions of the longitudinal axis of the hippocampus exhibit differential sensitivity to glucocorticoids. Mol. Psychiatry. 10.1038/s41380-020-0848-8 32709996

[B28] Li N. Lee B. Liu R.-J. Banasr M. Dwyer J. M. Iwata M. (2010). mTOR-Dependent Synapse Formation Underlies the Rapid Antidepressant Effects of NMDA Antagonists. Sci. (80-. ). 329, 959 LP–959964. 10.1126/science.1190287 PMC3116441 20724638

[B29] Lussier A. L. Caruncho H. J. Kalynchuk L. E. (2009). Repeated exposure to corticosterone, but not restraint, decreases the number of reelin-positive cells in the adult rat hippocampus. Neurosci. Lett. 460, 170–174. 10.1016/j.neulet.2009.05.050 19477232

[B30] Lussier A. L. Romay-Tallón R. J. Kalynchuk L. E. Caruncho H. J. (2011). Reelin as a putative vulnerability factor for depression: Examining the depressogenic effects of repeated corticosterone in heterozygous reeler mice. Neuropharmacology 60, 1064–1074. 10.1016/j.neuropharm.2010.09.007 20849864

[B31] Lussier A. L. Lebedeva K. Fenton E. Y. Guskjolen A. Caruncho H. J. Kalynchuk L. E. (2013). The progressive development of depression-like behavior in corticosterone-treated rats is paralleled by slowed granule cell maturation and decreased reelin expression in the adult dentate gyrus. Neuropharmacology 71, 174–183. 10.1016/j.neuropharm.2013.04.012 23608736

[B32] Ma Z. Zang T. Birnbaum S. G. Wang Z. Johnson J. E. Zhang C.-L. (2017). TrkB dependent adult hippocampal progenitor differentiation mediates sustained ketamine antidepressant response. Nat. Commun. 8, 1668. 10.1038/s41467-017-01709-8 29162814 PMC5698402

[B33] McCormack J. Korownyk C. (2018). Effectiveness of antidepressants. BMJ 360, k1073. 10.1136/bmj.k1073 29523598

[B34] Mendez-David I. David D. J. Deloménie C. Beaulieu J.-M. Gardier A. M. Hen R. (2020). A non-linear relation between levels of adult hippocampal neurogenesis and expression of the immature neuron marker doublecortin. bioRxiv 2020, 5.26.115873. 10.1101/2020.05.26.115873 37421207

[B35] Niu S. Yabut O. D’Arcangelo G. (2008). The Reelin signaling pathway promotes dendritic spine development in hippocampal neurons. J. Neurosci. 28, 10339–10348. 10.1523/JNEUROSCI.1917-08.2008 18842893 PMC2572775

[B36] Pazini F. L. Cunha M. P. Rosa J. M. Colla A. R. S. Lieberknecht V. Oliveira Á. (2016). Creatine, Similar to Ketamine, Counteracts Depressive-Like Behavior Induced by Corticosterone via PI3K/Akt/mTOR Pathway. Mol. Neurobiol. 53, 6818–6834. 10.1007/s12035-015-9580-9 26660117

[B37] Pham K. Nacher J. Hof P. R. McEwen B. S. (2003). Repeated restraint stress suppresses neurogenesis and induces biphasic PSA-NCAM expression in the adult rat dentate gyrus. Eur. J. Neurosci. 17, 879–886. 10.1046/j.1460-9568.2003.02513.x 12603278

[B38] Plumpe T. Ehninger D. Steiner B. Klempin F. Jessberger S. Brandt M. (2006). Variability of doublecortin-associated dendrite maturation in adult hippocampal neurogenesis is independent of the regulation of precursor cell proliferation. BMC Neurosci 7, 77. 10.1186/1471-2202-7-77 17105671 PMC1657022

[B39] Rivera-Baltanas T. Romay-Tallon R. Dopeso-Reyes I. G. Caruncho H. J. (2010). Serotonin transporter clustering in blood lymphocytes of reeler mice. Cardiovasc. Psychiatry Neurol. 2010, 396282. 10.1155/2010/396282 20414372 PMC2858282

[B40] Rivera-Baltanas T. Olivares J. M. Calado-Otero M. Kalynchuk L. E. Martinez-Villamarin J. R. Caruncho H. J. (2012). Serotonin transporter clustering in blood lymphocytes as a putative biomarker of therapeutic efficacy in major depressive disorder. J. Affect. Disord. 137, 46–55. 10.1016/j.jad.2011.12.041 22257570

[B41] Rivera-Baltanas T. Agis-Balboa R. C. Romay-Tallon R. Kalynchuk L. E. Olivares J. M. Caruncho H. J. (2015). Serotonin transporter clustering in blood lymphocytes predicts the outcome on anhedonia scores in naïve depressive patients treated with antidepressant medication. Ann. Gen. Psychiatry 14, 45. 10.1186/s12991-015-0085-8 26697099 PMC4687131

[B42] Romay-Tallon R. Kulhawy E. Brymer K. J. Allen J. Rivera-Baltanas T. Olivares J. M. (2018). Changes in Membrane Protein Clustering in Peripheral Lymphocytes in an Animal Model of Depression Parallel Those Observed in Naive Depression Patients: Implications for the Development of Novel Biomarkers of Depression. Front. Pharmacol. 9, 1149. 10.3389/fphar.2018.01149 30374301 PMC6196231

[B43] Shinohara K. Efthimiou O. Ostinelli E. G. Tomlinson A. Geddes J. R. Nierenberg A. A. (2019). Comparative efficacy and acceptability of antidepressants in the long-term treatment of major depression: protocol for a systematic review and networkmeta-analysis. BMJ Open 9, e027574. 10.1136/bmjopen-2018-027574 PMC6530313 31110100

[B44] Soumier A. Carter R. M. Schoenfeld T. J. Cameron H. A. (2016). New Hippocampal Neurons Mature Rapidly in Response to Ketamine But Are Not Required for Its Acute Antidepressant Effects on Neophagia in Rats. eNeuro 3, ENEURO.0116–15.2016. 10.1523/ENEURO.0116-15.2016 PMC4819285 27066531

[B45] Sterner E. Y. Kalynchuk L. E. (2010). Behavioral and neurobiological consequences of prolonged glucocorticoid exposure in rats: relevance to depression. Prog. Neuropsychopharmacol. Biol. Psychiatry 34, 777–790. 10.1016/j.pnpbp.2010.03.005 20226827

[B46] Teixeira C. M. Martín E. D. Sahún I. Masachs N. Pujadas L. Corvelo A. (2011). Overexpression of reelin prevents the manifestation of behavioral phenotypes related to schizophrenia and bipolar disorder. Neuropsychopharmacology 36, 2395–2405. 10.1038/npp.2011.153 21814183 PMC3194085

[B47] Thacker J. S. Yeung D. H. Staines W. R. Mielke J. G. (2016). Total protein or high-abundance protein: Which offers the best loading control for Western blotting? Anal. Biochem. 496, 76–78. 10.1016/j.ab.2015.11.022 26706797

[B48] Wong E. Y. H. Herbert J. (2006). Raised circulating corticosterone inhibits neuronal differentiation of progenitor cells in the adult hippocampus. Neuroscience 137, 83–92. 10.1016/j.neuroscience.2005.08.073 16289354 PMC2651634

[B49] Yuen E. Swanson S. Witkin J. M. (2017). Prediction of human efficacious antidepressant doses using the mouse forced swim test. Pharmacol. Biochem. Behav. 161, 22–29. 28888484 10.1016/j.pbb.2017.09.002

[B50] Zanos P. Moaddel R. Morris P. J. Georgiou P. Fischell J. Elmer G. II (2016). NMDAR inhibition-independent antidepressant actions of ketamine metabolites. Nature 533, 481–486. 10.1038/nature17998 27144355 PMC4922311

[B51] Zanos P. Moaddel R. Morris P. J. Riggs L. M. Highland J. N. Georgiou P. (2018). Ketamine and Ketamine Metabolite Pharmacology: Insights into Therapeutic Mechanisms. Pharmacol. Rev. 70, 621–660. 10.1124/pr.117.015198 29945898 PMC6020109

[B52] Zarate C. A. J. Singh J. B. Carlson P. J. Brutsche N. E. Ameli R. Luckenbaugh D. A. (2006). A randomized trial of an N-methyl-D-aspartate antagonist in treatment-resistant major depression. Arch. Gen. Psychiatry 63, 856–864. 10.1001/archpsyc.63.8.856 16894061

[B53] Zhou W. Wang N. Yang C. Li X.-M. Zhou Z.-Q. Yang J.-J. (2014). Ketamine-induced antidepressant effects are associated with AMPA receptors-mediated upregulation of mTOR and BDNF in rat hippocampus and prefrontal cortex. Eur. Psychiatry 29, 419–423. 10.1016/j.eurpsy.2013.10.005 24321772

